# Development of zeaxanthin‐rich tomato fruit through genetic manipulations of carotenoid biosynthesis

**DOI:** 10.1111/pbi.13387

**Published:** 2020-05-11

**Authors:** Uri Karniel, Amit Koch, Dani Zamir, Joseph Hirschberg

**Affiliations:** ^1^ Department of Genetics Alexander Silberman Institute of Life Sciences The Hebrew University of Jerusalem Jerusalem Israel; ^2^ Robert H. Smith Institute of Plant Sciences and Genetics The Hebrew University of Jerusalem Rehovot Israel

**Keywords:** zeaxanthin, carotenoid biosynthesis, metabolic engineering, antioxidants, AMD

## Abstract

The oxygenated carotenoid zeaxanthin provides numerous benefits to human health due to its antioxidant properties. Especially it is linked to protecting, together with the xanthophyll lutein, the retina in the human eye by filtering harmful blue light thus delaying the progression of age‐related macular degeneration (AMD), the most prevalent cause of blindness in developed countries. Despite its high nutritional value, zeaxanthin is less available than other substantial carotenoids in our diet. To solve this shortage, we chose to develop a new food source that would contain a high concentration of natural zeaxanthin. Tomato (*Solanum lycopersicum* L.) was selected as the target plant since it is the second largest vegetable crop grown worldwide and its fruit characteristically synthesizes and accumulates a high concentration of carotenoids. We employed two genetic approaches in order to enhance zeaxanthin biosynthesis in tomato fruit: a transgenic metabolic engineering and classical genetic breeding. A nontransgenic tomato line, named ‘Xantomato’, was generated whose fruit accumulated zeaxanthin at a concentration of 39 μg/g fresh weight (or 577 μg/g dry weight), which comprised ca. 50% of total fruit carotenoids compared to zero in the wild type. This is the highest concentration of zeaxanthin reached in a primary crop. Xantomato can potentially increase zeaxanthin availability in the human diet and serve as raw material for industrial applications.

## Introduction

Carotenoid pigments play indispensable functions in all photosynthetic organisms as accessory pigments for light harvesting, in the photosynthetic reaction centres and in protecting the photosynthetic apparatus against oxygen radicals produced by excessive light energy. Carotenoids are synthesized in plants, algae, cyanobacteria and certain fungi and nonphotosynthetic bacteria, but not in animals. Nonetheless, carotenoids are essential components of the human diet. Firstly and most importantly, β‐carotene, α‐carotene and β‐cryptoxanthin serve as the main precursors for the biosynthesis of vitamin A (retinol) and its derivative all‐*trans*‐retinoic acid. Other health benefits of carotenoids are related to their properties as antioxidants. They are implicated in reducing cancer and cardiovascular diseases and enhancing the immune system and bone health (Eggersdorfer and Wyss, 2018; Rodriguez‐Concepcion *et al.*, 2018). Furthermore, specific health benefits for brain development and cognitive functions have been linked to lutein (Giordano and Quadro, 2018; Johnson, 2014; Stringham *et al.*, 2019).

The xanthophylls (oxygenated carotenoids) zeaxanthin, lutein and their metabolite *meso*‐zeaxanthin, accumulate in the *macula lutea* of the human retina (Arunkumar *et al.*, 2020; Sauer *et al.*, 2019). In recent years, it has been recognized that these xanthophylls protect the macula by filtering damaging blue‐light irradiation and through their antioxidant activity (Arunkumar *et al.*, 2018; Johnson, 2014; Sauer *et al.*, 2019). High dietary intake of lutein and zeaxanthin, and elevated levels of these xanthophylls in the serum are associated with lower risk of age‐related macular degeneration (AMD) (Sabour‐Pickett *et al. *(2012), which is the most prevalent cause of blindness in developed countries (Congdon *et al.*, 2004; Flaxman *et al.*, 2017). Dietary supplementation of lutein and zeaxanthin decreased the progression of AMD by ~10% (AREDS^2^ Research Group, 2013; Chew *et al.*, 2013). Based on the AREDS2 nutritional recommendations, lutein and zeaxanthin are now the standard of care for reducing the probability of advanced AMD (Arunkumar *et al.*, 2018; Gorusupudi *et al.*, 2017; Sauer *et al.*, 2019; Wu *et al.*, 2015). Other health benefits of zeaxanthin include anti‐inflammatory effects (Johnson, 2014), reduced risk of atherosclerosis (Dwyer *et al.*, 2001), head and neck cancer (Leoncini *et al.*, 2016) and breast cancer (Wang *et al.*, 2014). Despite these benefits, zeaxanthin availability in our diet is relatively poor. Except for scallions, green leafy vegetables, which are rich in lutein, contain zero or negligible amounts of zeaxanthin (Perry *et al.*, 2009). Some fruits, such as peppers, oranges, papaya and mango, contain zeaxanthin within the range of 2–10 μg/g fresh weight (FW) (Dias *et al.*, 2018; Holden *et al.*, 1999; Perry *et al.*, 2009; Sajilata *et al.*, 2008). In specific varieties of paprika and orange peppers and Goji berries (*Lycium* fruit), zeaxanthin can reach 600–800 μg/g dry weight (ca. 45–70 μg/g FW) (Yossa Nzeuwa*et al.*, 2019; Burri *et al.*, 2016; Deli *et al.*, 1996; García *et al.*, 2007; Hornero‐Méndez *et al.*, 2002; Maiani *et al.*, 2009; Niro *et al.*, 2017; Patsilinakos *et al.*, 2018). However, these are high‐cost minor foods for most consumers. A common source of zeaxanthin in human nutrition is sweet corn with ca. 5 μg/g FW zeaxanthin in regular varieties although a higher concentration of up to 25 μg/g FW has been reported in kernels of bio‐fortified varieties (O'Hare *et al.*, 2015; O'Hare *et al.*, 2014). The concentration of zeaxanthin in the egg yolk, which is the major nonplant dietary source of xanthophylls, is 6–10 μg/g (Rasmussen *et al.*, 2012). It is therefore desirable to develop a low‐cost and sustainable source of natural zeaxanthin in a widespread crop that can serve the fresh market as well as industrial production. Tomato (*Solanum lycopersicum*) is globally the second largest vegetable crop with a yearly worldwide production of over 180 million tonnes (FAO, http://www.fao.org/faostat/en/?#data/QC). Tomato fruit synthesizes carotenoids that accumulate in chromoplasts, organelles adapted to store high concentration of carotenoids. Fresh tomato fruit contains 60–160 μg/g FW of lycopene, which constitutes 80%–95% of the total carotenoid content. These attributes make tomato an excellent target crop of metabolic engineering to produce zeaxanthin.

**Figure 1:**
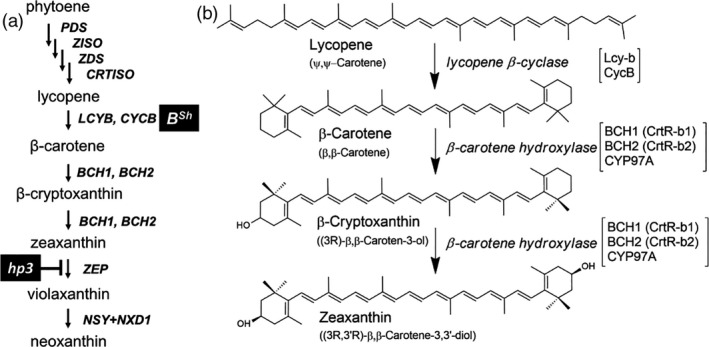
(a) The biosynthetic pathway of carotenoids, from geranylgeranyl diphosphate to β‐xanthophylls. (b) A detailed description of the conversion of lycopene to zeaxanthin in tomato. CycB, chromoplastic lycopene β‐cyclase; CrtR‐b (BCH), nonhaem β‐carotene hydroxylase; CRTISO, ζ‐carotene isomerase; CYP97B, cytochrome p450 β‐carotene hydroxylase; LcyB, chloroplastic lycopene β‐cyclase. NSY, neoxanthin synthase; NXD1, neoxanthin‐deficient 1; PDS, phytoene desaturase; PSY, phytoene synthase; ZDS, ζ‐carotene desaturase; ZEP, zeaxanthin epoxidase; *cycB*, (*B^sh^*), a dominant allele of *HIGH‐BETA*; *hp3*, A null mutation in ZEP.

Carotenoid biosynthesis in plants has been described at the molecular level (reviewed in (Hirschberg, 2001; Moise *et al.*, 2014; Rosas‐Saavedra and Stange, 2016; Ruiz‐Sola and Rodriguez‐Concepcion, 2012; Yuan *et al.*, 2015). This process takes place within plastids and catalysed by nuclear‐encoded enzymes (Figure 1a). Regulation of phytoene synthase (PSY) has been shown to determine the flux of the pathway, thus affecting the level of total carotenoids. Phytoene undergoes desaturation and geometrical isomerization to yield all‐*trans*‐lycopene. Cyclization of both ends of the linear molecule lycopene by lycopene β‐cyclase (LCYB) generates β,β‐carotene (β‐carotene), with two identical β‐ionone rings, whereas cyclization at one end by LCYB and the other end by ε‐cyclase (LCY‐E), yielding an ε‐ionone ring, produces α‐carotene (β,ε‐carotene). Beta‐carotene is converted to zeaxanthin following hydroxylation of both β‐rings. Three β‐ionone hydroxylase enzymes exist in tomato, the nonhaem iron BCH1 and BCH2, and the p450‐type hydroxylase CYP97A (Figure 1b). The enzyme zeaxanthin epoxidase (ZEP) converts zeaxanthin to violaxanthin that is metabolized to neoxanthin by a yet unidentified neoxanthin synthase (NSY).

The fleshy fruit of tomato develops from ovary over a period of 6–8 weeks after anthesis. During this period, the fruit is green and its carotenoid composition is comparable to leaves, comprising of mainly photosynthesis‐associated xanthophylls. At the onset of ripening, also known as the ‘breaker’ stage, lycopene begins to accumulate due mainly to changes in gene expression. At this stage, transcription of genes for lycopene synthesis is up‐regulated while transcription of genes for both lycopene ε‐cyclase (LCY‐E) and lycopene β‐cyclase (LCYB), which metabolize lycopene, is down‐regulated (Bramley, 2002; Enfissi *et al.*, 2017; Pecker *et al.*, 1996; Ronen *et al.*, 2000; Ronen *et al.*, 1999). Expression of these genes is developmentally regulated but also affected by other factors, such as environment and hormones (Cruz *et al.*, 2018; Giovannoni *et al.*, 2017; Klee and Giovannoni, 2011; Li *et al.*, 2019; Liu *et al.*, 2015; Sun and Li, 2020).

Over the last two decades, metabolic engineering of carotenoid biosynthesis in crop plants has been employed in order to increase their nutritional and economic value (reviewed in (Giuliano, 2014; Giuliano, 2017; Watkins and Pogson, 2020; Zheng *et al.*, 2020). Tomato fruit contains a high concentration of lycopene as the major carotenoid pigment that accumulates during ripening due to its enhanced synthesis, diminished metabolism to cyclic carotenes and sequestration in a crystalline form. These metabolic processes are predominantly regulated by differential gene expression. Several attempts to manipulate the expression of lycopene β‐cyclase and β‐carotene hydroxylase genes to facilitate xanthophyll accumulation in tomato fruit have been previously reported (D'Ambrosio *et al.*, 2011; D'Ambrosio *et al.*, 2011; Enfissi *et al.*, 2019; Giorio *et al.*, 2008; Giuliano, 2014; Giuliano *et al.*, 2008; Huang *et al.*, 2013; Nogueira *et al.*, 2017). Concurrent overexpression of carotene beta‐hydroxylase 1 (BCH1, CrtR‐b1) and lycopene β‐cyclase (LCYB) did not result in any appreciable increase in the xanthophyll content of ripe fruits compared to the overexpression of only the LCYB transgene (Giorio *et al.*, 2008). Overexpression of carotene beta‐hydroxylase 2 (BCH2, CrtR‐b2) in tomato fruit produced free violaxanthin and a significant amount of esterified xanthophylls in hemizygous transgenic plants at the immature green stage (D'Ambrosio *et al.*, 2011). Yet, at the ripe stage, total xanthophyll content was only 8% of total carotenoids with small amounts of zeaxanthin (D'Ambrosio *et al.*, 2011). However, another experiment succeeded to obtain ca. 13 μg/g FW of zeaxanthin plus β‐cryptoxanthin in tomato fruit by simultaneous transgenic expression of Arabidopsis LCYB and pepper BCH (b‐Chy) genes with the Pds promoter (Dharmapuri *et al.*, 2002).

Here, we describe the development of tomato varieties whose fruit synthesize and accumulate zeaxanthin as their main carotenoid constituent. To this end, we have utilized existing mutations that affect carotenoid biosynthesis in a classic genetic breeding approach as well as a transgenic metabolic engineering of carotenoid biosynthesis in fruit.

## Results

### Genetic breeding of high‐zeaxanthin tomato

**Figure 2:**
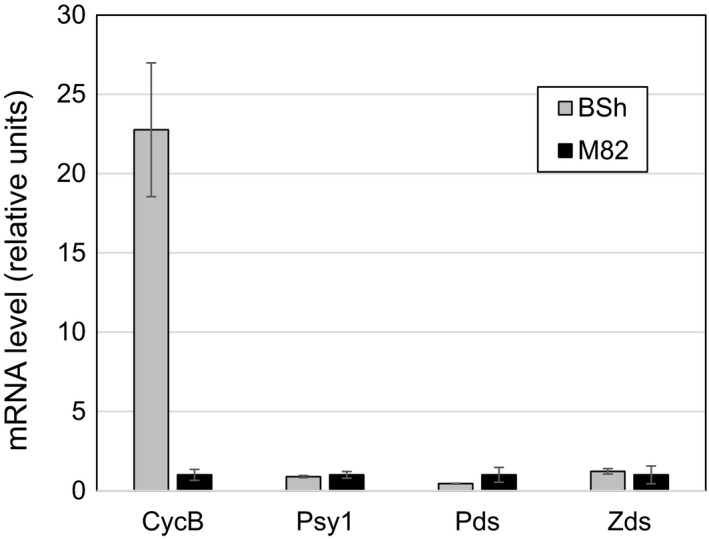
Expression of carotenoid biosynthesis genes in ripe fruits (breaker + 3 days) of wild type (M82) and *B^Sh^* lines. Gene annotations as in Figure 1. Messenger RNA was quantified by determined by real‐time polymerase chain reaction (PCR) as described in Experimental Procedures.

Tomato fruit mainly accumulates lycopene as a carotenoid end product due to the down‐regulation of expression of Lcy‐e and Lcy‐b (Hirschberg, 2001; Pecker *et al.*, 1996; Ronen *et al.*, 1999). The first step towards obtaining beta‐xanthophylls in tomato fruit is enhancing β‐carotene synthesis. It was previously established that a chromoplast‐specific lycopene β‐cyclase, CYCB, is responsible for β‐carotene synthesis in tomato fruit (Ronen *et al.*, 2000). Several dominant alleles of *HIGH‐BETA* were isolated following the introgression of the CycB (*B*) gene from wild tomato species (Tomes *et al.*, 1956). We have characterized an allele of *HIGH‐BETA* in the heirloom tomato line ‘Jaune Flamme’ of an unknown genetic background with an indeterminate growth habit and orange‐coloured fruit. The genomic DNA sequence of CycB from this line was found to be identical to the gene from the green‐fruited wild species *Solanum habrochaites* (LA0316) (Dalal *et al.*, 2010) (The C.M. Rick Tomato Genetics Resource Center, TGRC, https://tgrc.ucdavis.edu/index.aspx), which was previously described (NCBI Accession KP233161 (Orchard, 2014). Therefore, we termed this allele *B^Sh^*. Fruits of *B^Sh^* accumulated β‐carotene up to 80% of total carotenoids (Table 1). To verify that accumulation of β‐carotene was linked to CycB, we crossed mutant *B^Sh^* with the ‘wild‐type’ tomato variety M82. The high β‐carotene phenotype was co‐segregated with the *B^Sh^* allele in F2 offspring at a ratio of 1:3. The intermediate concentration of β‐carotene in the fruit of F1 hybrid of *B^Sh^*xM82 compared with parental line *B^Sh^* fits the semi‐dominance nature of the allele (Table 1). Expression of *CycB* in *B^Sh^* during ripening, measured by qRT‐PCR, was 40‐fold higher than in the wild‐type variety M82, while expression of other genes in the carotenoid biosynthesis pathway, such as Pds, Zds and Psy1, did not significantly change (Figure 2).

**Table 1:** Carotenoid concentration in ripe fruits (μg/g fresh weight, FW)

Line/ Genotype	Phytoene + Phytofluene	Lycopene	β‐Carotene	β‐Cryptoxanthin	Zeaxanthin	Lutein	Others	Total carotenoids	Per cent of β‐xanth
M82	8.8 ± 2.8	45.8 ± 7.3	1.0 ± 0.2		0.1 ± 0.1	0.4 ± 0.2	0.8	56.9 ± 10.1	<1
*B^sh^*s*	0.9 ± 0.4	2.2 ± 0.7	22.4 ± 1.8	0.1 ± 0.1		0.4 ± 0.2	1.4	27.3 ± 2.0	<1
*B^Sh^*x M82 (F1)	3.0 ± 0.8	16.7 ± 2.5	15.4 ± 2.0	0.1		0.7 ± 0.1	2.5^†^	38.3 ± 4.4	<1
*hp3*	6.3 ± 1.2	26.6 ± 6.7	3.5 ± 0.4		5.2 ± 0.3	2.7 ± 0.9	0.5	44.7 ± 7.6	11.6
*Green‐stripe (gs)*	7.6 ± 1.2	78.6 ± 13.8	11.8 ± 0.8			1 ± 0.1		99.1 ± 13.7	
*Green‐flesh (gf)*	4.2 ± 1.4	25.6 ± 8.4	2.9 ± 0.7			2.1 ± 0.2	1.7	33.9 ± 9.3	0
*hp3*/*B^Sh^*	1.0 ± 0.3	1.6 ± 0.8	26.1 ± 3.5	0.3 ± 0.1	7.7 ± 0.6	1.1 ± 0.6	0.3	38.5 ± 4.4	20.8
*hp2^dg^*	29.0 ± 2.4	105.1 ± 4.9	9.4 ± 0.3					148.1 ± 4.1	
*hp3*/*gf*	8.5 ± 1.5	81.3 ± 7.2	7.9 ± 1.3		7.8 ± 0.5	3.1 ± 0.3	0.2	108.8 ± 8.3	7.2
*hp3*/*gs*	9.6 ± 0.4	34 ± 1.0	2.2 ± 0.3		4.2 ± 0.2	0.6 ± 0.1		50.6 ± 3.8	8.3
*hp3/B^Sh^/gf*	3.3 ± 0.8	1.8 ± 1.2	33.8 ± 6.0	0.5 ± 0.2	7.0 ± 1.4	0.7 ± 0.1	4.5^‡^	53.3 ± 7.6	14.1
*hp3/B^Sh^/gs*			24.3 ± 3.7	1.0 ± 0.1	16.3 ± 2	0.6 ± 0.3		42.6 ± 3.9	40.6
*hp3/B^Sh^/gs/hp2^dg^* #1 (F2)	1.0 ± 0.1	0.1	26.7 ± 0.3	0.6	34.8 ± 0.8	1.5 ± 0.2	0.5	65.5 ± 1.4	53.7
*hp3/B^Sh^/gs/hp2^dg^* #2 (F2)	2.2 ± 0.3	0.8	37.2 ± 2.2	1.1 ± 0.1	32.4 ± 2.4	0.9 ± 0.1	0.8	75.7 ± 4.2	44.4
*hp3/B^Sh^/gs/hp2^dg^* #3 (F2)	0.8 ± 0.1		21.4 ± 2.4	0.6	22.6 ± 0.8	0.6 ± 0.3	1.6	47.5 ± 3.8	48.8
*hp3/B^Sh^/gs/hp2^dg^* #4 (F2)	1.8 ± 0.5		34.0 ± 2.2	0.8	25.4 ± 2.2	1.4 ± 0.5	1.2	64.5 ± 4.5	40.6
*hp3/B^Sh^/gs/hp2^dg^* #5 (F3)	4.7 ± 0.6		34.7 ± 4.9	0.8 ± 0.2	37.8 ± 3.5	2.3 ± 0.6		80.4 ± 9.7	48.0
*hp3/B^Sh^/gs/hp2^dg^* #6 (F3)	3.9 ± 0.2		37.0 ± 1.9	1.0 ± 0.1	39.0 ± 0.7	1.7 ± 0.2		82.6 ± 2.6	48.4
*hp3/B^Sh^/gs/hp2^dg^* #7 (F3)	6.2 ± 2		43.4 ± 5.2	0.7 ± 0.1	33.5 ± 4.6	3.9 ± 1		87.8 ± 10.1	39.0

^*^ Jaune Flamme line of unknown genetic background.

^†^ γ‐carotene 2.2.

^‡^ γ‐carotene 3.7.

Mutant *B^Sh^* was crossed with *high‐pigment 3* (*hp3*), a tomato mutant impaired in zeaxanthin epoxidase (ZEP) (Galpaz *et al.*, 2008). Both leaves and fruit of *hp3* contained significantly higher amounts of zeaxanthin (Tables 1 and 2). Accumulation of zeaxanthin in the fruit of the double‐mutant *B^Sh^/hp3* was approximately 50% higher than in the single mutant *hp3* (Table 1). While the conversion of lycopene to β‐carotene was more efficient in *B^Sh^*/*hp3* plants, only a small fraction of β‐carotene was utilized for the synthesis of β‐xanthophylls.

**Table 2:** Carotenoid composition in tomato leaves (μg/g FW)

Line	β‐Carotene	Zeaxanthin	Antheraxanthin	Violaxanthin	Neoxanthin	Lutein	Total carotenoids
M82	6.1 ± 0.4	0.8 ± 0.2	1.1 ± 0.1	22.7 ± 2.6	32.7 ± 2.3	82.1 ± 6.3	145.5 ± 11
*CrtI*	5 ± 1.4	1.6	3.6 ± 0.4	24.2 ± 3.1	19.8 ± 1.0	38.9 ± 0.6	93.1 ± 3.9
*hp3*	2.4 ± 0.3	27.3 ± 3.6	4.1 ± 0.9		3.7 ± 0.9	51.7 ± 14.2	89.2 ± 13.8
*hp3/B^Sh^/wf/CcBCH2#1*	2.1 ± 0.9	26.5 ± 4.8	12.1 ± 3	2.7 ± 0.9	16.7 ± 2.8	22 ± 2.3	82.2 ± 13.3

To increase xanthophyll synthesis, we crossed the double‐mutant *B^Sh^/hp3* with two different recessive mutants: the green fruit mutant *STAY‐GREEN*, which is impaired in the SlSGR1 protein (Luo *et al.*, 2013) and *GREEN‐STRIPE* (*gs*). The rationale behind these crossings was inspired by observations suggesting that fruits of various green‐flesh varieties contain a higher concentration of total carotenoids, including xanthophylls incorporated in the photosynthetic apparatus (Luo *et al.*, 2013). The concentration of zeaxanthin in fruits of the triple mutant *B^Sh^/hp3/gs* more than doubled compared the double‐mutant *B^Sh^/hp3*, to a significant level of 16 µg/g FW (Table 1, Figure S1).

**Figure 3:**
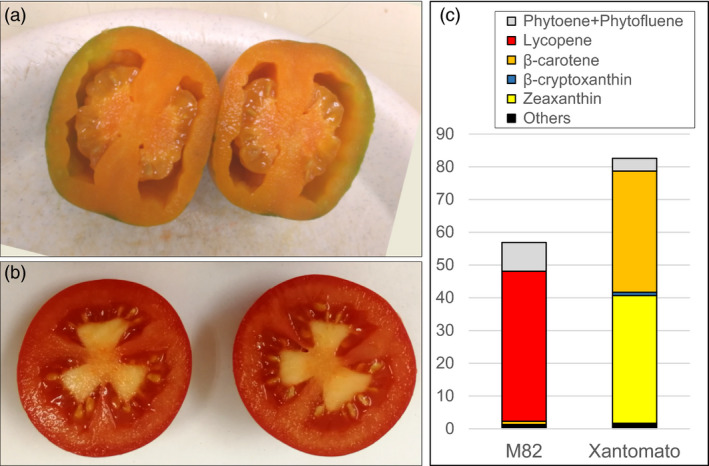
(a) Typical appearance of zeaxanthin‐containing fruit of the genotype *B^Sh^/hp3/gs/hp2^dg^* (Xantomato); (b) fruit of the variety M82 (b); (c) carotenoid composition in Xantomato and WT fruit (μg/g FW).

The triple mutant *B^Sh^/hp3/gs* line was crossed with the *high‐pigment* mutant *hp2^dg^* (Levin *et al.*, 2006) to generate a quadruple homozygous mutant *B^Sh^/hp3/gs/hp2^dg^*, which was confirmed by genotyping of F2 plants. The recessive mutation *hp2* in the DE‐ETIOLATED 1 (DET1) gene (Mustilli *et al.*, 1999) was found to increase the plastid compartment size in leaf and fruit cells thus elevating chlorophyll levels in immature fruit and total carotenoids in ripe fruit (Azari *et al.*, 2010). The carotenoid composition of four lines from this F2 population exhibited a high level of zeaxanthin accumulation up to 34.8 µg/g FW (Table 1, Figure S1) and their typical appearance is presented in Figure 3 and Figure S2. Zeaxanthin concentration in F3 offspring of self‐pollinated line *hp3/B^Sh^/gs/hp2^dg^* #1 reached 39.0 µg/g FW, which equal to 557 µg/g in dry weight (DW) (Table 1). This line, which also contained 37 µg/g FW (529 µg/g DW) β‐carotene, was named ‘Xantomato’.

**Figure 4:**
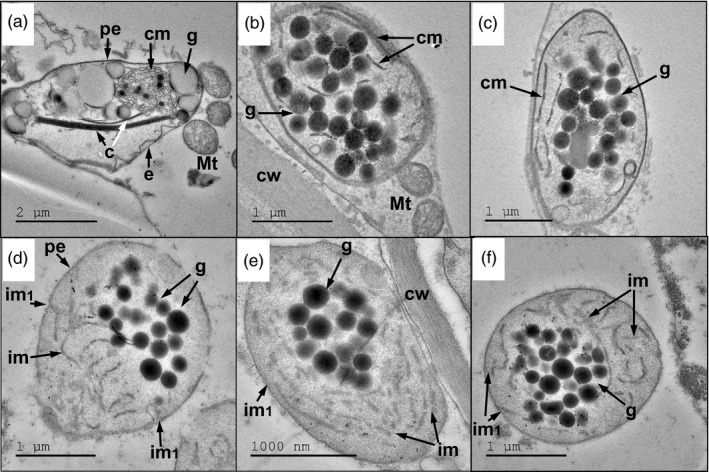
Electron micrographs of chromoplasts in pericarp cells of ripe fruits. (a) M82, ‘wild type’ with mainly lycopene (80%–85% of total carotenoids); (b, c) *B^Sh^*, with 80%–85% β‐carotene; (d–f) *B^Sh^/hp3/gs/hp2^dg^*, with 40% β‐carotene and 53% zeaxanthin. c—lycopene crystals; cm—crystalline membranes; cw—cell wall; g—globules; im—rod‐shaped internal membranes; im1—developing rod‐shaped internal membranes; pe—plastid envelope; bars indicate actual size.

The ultrastructure of chromoplasts in the ripe fruit of *HIGH‐BETA* (*B^Sh^*) and the zeaxanthin‐accumulating quadruple mutant *B^Sh^/hp3/gs/hp2^dg^* was examined using transmission electron microscopy (TEM) (Figure 4). Lycopene present in the chromoplasts of tomato fruit is sequestered in the form of crystalline structures (Harris and Spurr, 1969a; Rosso, 1968), but other carotenoids can be stored in different types of formations (Harris and Spurr, 1969b; Mohr, 1979; Nogueira *et al.*, 2013). The plastids in M82 fruit, which mainly accumulated lycopene and phytoene, contained typical crystals and remnant lamella membranes with crystalline structures and a few large plastoglobuli (Figure 4a). Beta‐carotene‐accumulating plastids in *B^Sh^* fruit contained many plastoglobuli accompanied by a small number of rod‐shaped crystalline membranes (Figure 4b,c). The chromoplasts of the quadruple mutant *B^Sh^/hp3/gs/hp2^dg^*, which contained approximately equal amounts of β‐carotene and zeaxanthin, encompassed fewer plastoglobuli than in *B^sh^*. In addition, these chromoplasts contained many rod‐shaped membranous structures (Figure 4d–f). Since this phenomenon correlates with the accumulation of xanthophylls, it is likely that β‐carotene is mainly stored in plastoglobuli while zeaxanthin is sequestered in the rod‐shaped membranous structures.

### Transgenic breeding of high‐zeaxanthin tomato

**Figure 5:**
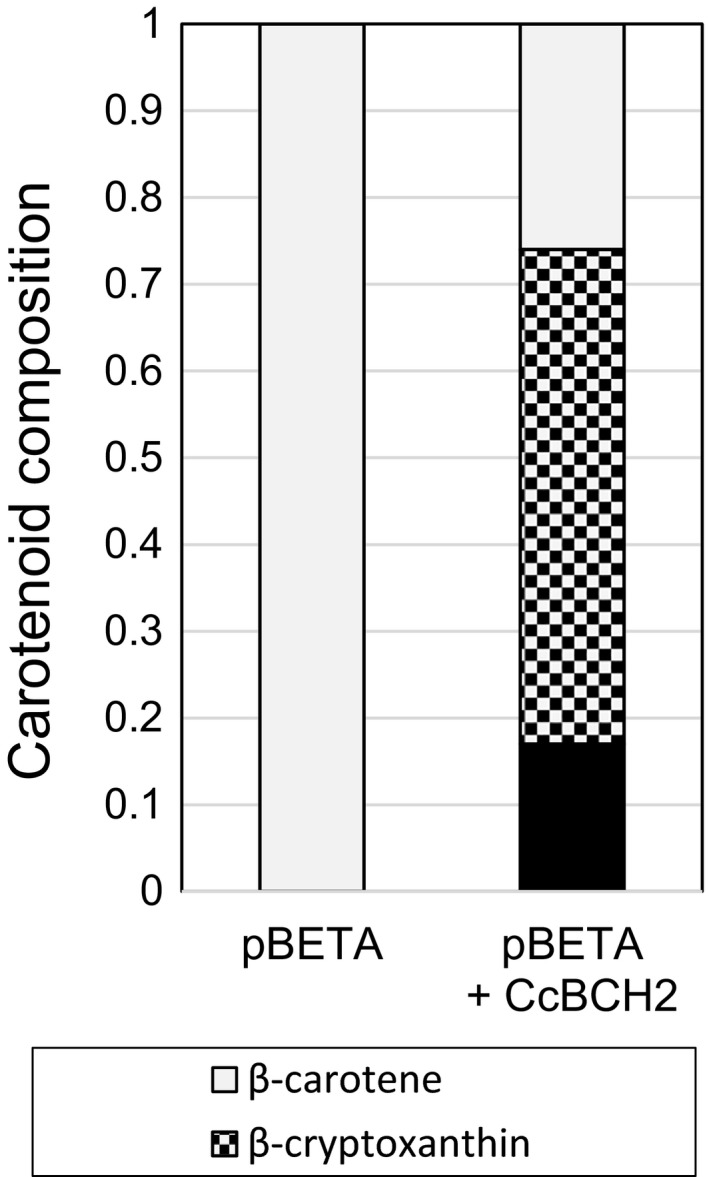
Carotenoid composition in *E. coli* cells expressing BCH2 from *C. clementina*. Total carotenoids from *E. coli* cells carrying the plasmid pBETA only contain β‐carotene. Cells containing both pBETA and CcBCH2 accumulate β‐cryptoxanthin and zeaxanthin.

An alternative approach to enhancing the hydroxylation of β‐carotene in tomato fruit is utilizing transgenic expression of β‐carotene hydroxylase (BCH). Considering the high levels of β‐xanthophylls that accumulate in *Citrus*, we chose the BCH2 gene from *Citrus clementina*. Fruits of this species also accumulate a significant amount of β‐cryptoxanthin (Dhuique‐Mayer *et al.*, 2005). Full‐length cDNA of BCH2 from *Citrus clementina* without the first 89 codons that encode the predicted transit peptide, in the plasmid vector pBCH2 was expressed in β‐carotene‐producing cells of *E. coli* that carried plasmid pBETA (Cunningham and Gantt, 2007). Carotenoid analysis confirmed that the *C. clementina* BCH2 (CcBCH2) exhibited β‐carotene hydroxylation activity in *E. coli* (Figure 5). The level of zeaxanthin in *E. coli* was lower than that of β‐cryptoxanthin, indicating that β‐carotene di‐hydroxylation by CcBCH2 in *E. coli* cells is less efficient than the mono‐hydroxylation (Figure 5).

Next, the *C. clementina* BCH2 full‐length cDNA behind the constitutive CaMV 35S promoter was introduced into the *hp3/B^Sh^* double‐mutant tomato plant using Agrobacterium‐mediated transformation. Seven independent transgenic plants of *hp3/B^Sh^/35S:CcBCH2* were obtained. A number of the transgenic plants exhibited slightly yellow fruit at the mature‐green stage due to an accumulation of zeaxanthin already present at that stage (Table 3)*.* Carotenoid composition in the ripe fruit of T1 plants revealed a wide range of xanthophyll accumulation in different transgenic lines (Table 3). Zeaxanthin made up the majority of β‐xanthophylls accumulated with only a small amount of β‐cryptoxanthin present. The transgenic line that accumulated the highest level of xanthophylls, *hp3/B^Sh^‐CcBCH2#1* (Table 3 and Figure S3), was selected for further breeding in this project.

**Table 3:** Carotenoid concentration (μg/g FW) in ripe fruit of transgenic tomato plants

Line	Phytoene + Phytofluene	Lycopene	β‐Carotene	β‐Cryptoxanthin	Zeaxanthin	Lutein	Others	Total carotenoids	Per cent of β‐xanth.
*hp3/B^Sh^*	1.0 ± 0.3	1.6 ± 0.8	26.1 ± 3.5	0.3 ± 0.1	7.7 ± 0.6	1.1 ± 0.6	0.3	38.5 ± 4.4	26.0
*hp3/B^Sh^/CcBCH2#1*	0.1 ± 0.1	0.1 ± 0.1	2.0 ± 0.4	0.5 ± 0.1	10.6 ± 1.1	0.5 ± 0.1		14.1 ± 1.6	78.7
*hp3/B^Sh^/CcBCH2#3*	3.5 ± 0.8	3.2 ± 0.6	24.7 ± 3.2		2.0 ± 0.3	0.3 ± 0.1	0.9	36.1 ± 3.8	0.1
*hp3/B^Sh^/CcBCH2#4*	5.0 ± 2.5	3.6	48.9 ± 6.2		2.2	2.1	1.6*	57.3	<0.1
*hp3/B^Sh^/CcBCH2#5*			0.41 ± 0.1	0.2	3.6 ± 0.6			4.2 ± 0.8	90.4
*hp3/B^Sh^/CcBCH2#6*			7.8 ± 0.8	0.5 ± 0.1	6.2 ± 0.9	0.1 ± 0.1		14.6 ± 1.6	45.9
*hp3/B^Sh^/CcBCH2#7*			2.1 ± 0.8	0.3 ± 0.1	4.2 ± 1.0			7.5 ± 1.6	60.0
*hp3/B^Sh^/CcBCH2#9*			26.1 ± 9.1		3.1 ± 0.4	0.7 ± 0.2	0.9	32.1 ± 12	0.1
*hp3/B^Sh^/CcBCH2#1/CrtI*			26.4 ± 7.2	0.6 ± 0.1	16.3 ± 1.5	1.9 ± 0.4	2.2	47.4 ± 8.5	35.7
*wf* ^†^		35 ± 5.2	1.7 ± 1.5			0.7 ± 0.1		42.2	0
*hp3/wf*	14.0 ± 1.1	29 ± 5.2			2.2 ± 0.1	2.1 ± 0.1		53.8 ± 3.8	<0.1
*hp3/B^Sh^/wf*	3 ± 0.3		49.2 ± 4.8	0.4 ± 0.4	13.6 ± 0.2	1.3 ± 0.1		67.5 ± 4.9	20.7
*hp3/B^Sh^/wf/CcBCH2#1*			2.6 ± 0.3	1.2 ± 0.3	22.6 ± 3.9	1.5 ± 0.3		28.9 ± 4.2	82.4

^*^ From Galpaz *et al. *(2006).

^†^ Mainly γ‐carotene.

To eliminate possible adverse effects due to the co‐suppression of expression by the endogenous β‐carotene hydroxylase, the transgenic line *hp3/B^Sh^/CcBCH2#1* was crossed with the mutant *white‐flower* (*wf*), which is impaired in the chromoplast‐specific β‐carotene hydroxylase, BCH2 (CrtR‐b2) (Galpaz *et al.*, 2006). The fruits of the homozygous triple mutant *hp3/B^Sh^/wf* contained a significantly higher concentration of zeaxanthin than those of *hp3/B^Sh^* (Tables 1 and 3).

The triple mutant *hp3/B^Sh^/CcBHC2#1* was crossed with a transgenic tomato line overexpressing the bacterial phytoene desaturase gene, *crtI*, under the control of the constitutive CaMV *35S* promoter (Romer *et al.*, 2000). The concentrations of the xanthophylls in *hp3/B^Sh^/C.hx‐1/CrtI* fruit increased compared with *hp3/B^Sh^/CcBCH2#3*, with a significant increase in the β‐carotene levels (Tables 3 and 4; Figure S3)*.* This result points out the possibility that pushing the flux of the carotenoid biosynthetic pathway forward with overexpression of upstream enzymes could contribute to further elevation of zeaxanthin accumulation.

**Table 4:** Carotenoid composition in pericarp tissue of mature‐green fruit (μg/g FW)

Line	β‐Cryptoxanthin	Zeaxanthin	Violaxanthin	Neoxanthin	Lutein	Total carotenoids
M82			0.6 ± 0.1	0.4 ± 0.1	1.3 ± 0.2	2.2 ± 0.4
*hp3/B^Sh^/CcBCH2#1*		7.5			1.2 ± 0.1	8.7 ± 0.1
*hp3/B^Sh^/CcBCH2#1/CrtI*	0.2	14.5 ± 0.5			2.2 ± 0.2	16.8 ± 0.7

## Discussion

Metabolic engineering of zeaxanthin has been previously achieved in several plants. In potato (*Solanum tuberosu*m L.), concentration of zeaxanthin in tubers was tripled to 0.99 μg/g DW (3–5 μg/g FW) by silencing of LCY‐E (Diretto *et al.*, 2007) and to 40 μg/g dry weight (ca. 10 μg/g FW) by silencing of zeaxanthin epoxidase (ZEP) (Romer *et al.*, 2002). Transgenic expression in a white corn variety of Psy1 from corn, crtI from the bacteria *Pantoea ananatis* and Lcy‐b and Bch from *Gentiana lutea* increased dramatically zeaxanthin levels in seeds to 34.5 μg/g dry weight (Zhu *et al.*, 2008). In a yellow endosperm corn variety, transgenic expression of Psy1, crtI and Lcy‐b quadrupled zeaxanthin in seeds to 56.5 μg/g dry weight (Naqvi *et al.*, 2011). In tomato, accumulation of ca. 13 μg/g FW of zeaxanthin plus β‐cryptoxanthin in fruit was achieved by the transgenic expression of LCYB and BCH (Dharmapuri *et al.*, 2002). In this study, we have demonstrated that carotenoid biosynthesis in tomato fruit can be successfully manipulated nontransgenically to achieve zeaxanthin accumulation at a concentration of 39 μg/g FW (equal to 650 μg/g dry weight), which comprises ca. 50% of total fruit carotenoids. To the best of our knowledge, this is the highest level of zeaxanthin reached in a major crop such as tomato.

We ascribe the high‐zeaxanthin accumulation in Xantomato to the unique combination of three primary mutations: *HIGH‐BETA* (*B^sh^*), *high‐pigment 3* (*hp3*) and *green‐stripe* (*gs*) that transform an existing biosynthesis pathway dedicated to carotenoid accumulation in fruit. Fruit of the tomato mutant *HIGH‐BETA* accumulates β‐carotene due to high expression of the fruit‐specific lycopene β‐cyclase (CycB) (Ronen *et al.*, 2000). All known *HIGH‐BETA* (*B*) alleles were originated through genetic introgression from various green‐fruited wild tomato species (Lincoln and Porter, 1950; Ronen *et al.*, 2000; Stommel, 2001; Stommel and Haynes, 1994; Tomes *et al.*, 1958; Tomes *et al.*, 1954). We have used the allele *B^Sh^*, which was originated from *S. habrochaites*, because it displays the strongest phenotype among known *HIGH‐BETA* mutations as evident by the fact that β‐carotene in its fruits represents> 80% of total carotenoids (Table 1). This reflects a highly efficient lycopene cyclization activity by CYCB encoded by *B^Sh^*. The conversion of β‐carotene to downstream xanthophylls in tomato fruit was found to be more complicated to attain. Based on earlier studies, it has been suggested that the β‐carotene accumulated naturally in *HIGH‐BETA* fruit is not as accessible to hydroxylation by transgenic β‐hydroxylases as β‐carotene produced by a transgenic LCYB cyclase, possibly due to metabolic channelling between LCY‐b and BCY hydroxylase (Giuliano *et al.*, 2008).

It is apparent from our data that the total carotenoid concentration in *B^Sh^* fruit is lower than in wild‐type tomato. The steady‐state concentration of carotenoids in fruit is controlled by the flux of the biosynthetic pathway, through sequestration mechanisms that determine the storage capacity of the plastids, and by degradation processes that are catalysed by carotenoid cleavage dioxygenases (CCD) or by nonspecific cleavage facilitated by the lipoxygenase *TomLoxC* (Gao *et al.*, 2019). Loss of function of the enzyme zeaxanthin epoxidase (ZEP) in the mutant *hp3*, or silencing of the ZEP gene, causes a modest build‐up of zeaxanthin in fruit (Galpaz *et al.*, 2008; Romer *et al.*, 2002; Wolters *et al.*, 2010; Table 2). This result demonstrates that β‐carotene hydroxylation does take place to some extent in tomato fruit. Since zeaxanthin does not accumulate in either normal or *HIGH‐BETA* fruit, we deduce that it is metabolized to violaxanthin, which is the precursor for abscisic acid (ABA) synthesis. In addition, it is probable that fruit chromoplasts do not possess a mechanism to store free zeaxanthin, and thus, it can be constantly degraded by the CCD enzymes. Both processes cited above may explain the reduced quantity of total carotenoids in *B^Sh^* fruit.

While plants with a combination of *B^Sh^* and *hp3* mutations yielded fruit with 7.7 μg/g fresh weight of zeaxanthin, the addition of the *green‐stripe* (*gs*) genotype, more than doubled this concentration, and the ratio of zeaxanthin plus β‐cryptoxanthin to total β‐cyclized carotenoids increased from 27% to 41% (Table 1). The molecular nature of *gs* is yet unknown and, therefore, an explanation of its contribution to overall zeaxanthin accumulation is difficult. However, it appears that hydroxylation of β‐carotene in fruits is catalysed by the chloroplastidial enzymes BCH1 and/or CYP97A since the inactivation of the chromoplast‐specific nonhaem diiron enzyme BCH2 in the mutant *white‐flower* (Galpaz *et al.*, 2006) did not eliminate the biosynthesis of zeaxanthin (Table 3). Therefore, we suggest that *gs* elevates either the expression or activity of these enzymes. Furthermore, there remains a possibility that the *gs* mutation may somehow alter the mechanism in which zeaxanthin is degraded after the transition from chloroplast to chromoplast. By contrast, the allele *Green‐flesh* (*gf*), which elevated zeaxanthin level in *hp3* by 50%, did not show any effect in the *hp3*/*B^Sh^* double mutant (Table 1). This difference from *gs* can be explained by the different molecular basis of the green phenotype appearance of the fruit, which excludes the expression CYCB in the same cells where endogenous chloroplast‐type hydroxylases are active. The differences in fruit carotenoids among F2 plants of the genotype *hp3*/*B^Sh^*/*gs*/*hp2^dg^* (Table 1) reflect the genetic variability resulting from mixing four distinct tomato germplasms. It is expected that other alleles also affect zeaxanthin accumulation in the chromoplasts. Independent assortment of alleles in F2 plants has enabled the selection of quantitative trait loci (QTLs) for high zeaxanthin in F3 and can be further exploited in future genetic breeding.

The final increase of zeaxanthin accumulation was provided by the mutation *high‐pigment 2* (*hp2*), which impairs the tomato *DET1* homolog that functions in photomorphogenesis (Mustilli *et al.*, 1999) and enhances fruit metabolites (Liu *et al.*, 2004). Fruit of allele *hp2^dg^* of this mutation was found to be more active metabolically (Bino *et al.*, 2005; Kolotilin *et al.*, 2007; Levin *et al.*, 2006; Liu *et al.*, 2004). The combination of the alleles *hp3*, *B^Sh^*, *gs* and *hp2^dg^*, not only elevated the level of zeaxanthin but also raised its ratio to 49% of total fruit carotenoid content.

Xanthophylls found in the chromoplast of flowers are usually sequestered in the form of xanthophyll esters (Ariizumi *et al.*, 2014; Yamamizo *et al.*, 2010). This phenomenon was demonstrated also within fruit chromoplasts of transgenic tomatoes that accumulated ketocarotenoids that were mainly sequestered in esterified forms within plastoglobuli (Enfissi *et al.*, 2019). The entirety of the zeaxanthin that accumulated in the fruits examined over the course of our study was observed in its free form, that is nonesterified. Its sequestration in fruit is correlated with the appearance of rod‐shaped membrane structures observed in chromoplasts of the quadruple homozygous mutants but not in other mutants, including in the β‐carotene accumulating *B^Sh^* (Figure 4). In bell pepper chromoplasts, xanthophylls, including zeaxanthin, accumulate in specific lipoprotein fibrils that also contain galactolipids, phospholipids and fibrillin protein (Deruere *et al.*, 1994). The uncharacteristic rod‐shaped internal membranes seen in zeaxanthin‐containing chromoplasts can potentially sequester zeaxanthin. In bilayer membranes, free *trans*‐zeaxanthin adopts a transmembrane orientation with a tilt angle of ~ 40 degrees relative to the membrane plane with the polar hydroxyl groups located at the opposite polar zones of the lipid bilayer (Grudzinski *et al.*, 2017). It is possible that this form of sequestration limits the storage capacity of zeaxanthin in tomato chromoplast and free zeaxanthin molecules that are not associated with membranes are more susceptible to enzymatic degradation. Moreover, the two forms of carotenoid sequestration in chromoplasts, where β‐carotene is in plastoglobuli and zeaxanthin is in rod‐shaped membranes (Figure 4), can explain the limitation of converting all of the β‐carotene to zeaxanthin in the fruit of both *hp3/B^Sh^/gs* and *hp3/B^Sh^/CcBCH2*. Once β‐carotene is captured within plastoglobuli, it is less accessible to enzymatic hydroxylation. In *B^Sh^* fruit, part of the β‐carotene produced by the efficient cyclization activity of CYCB is sequestered in plastoglobuli, which are inaccessible to hydroxylases. This may also explain previous observations that β‐carotene in *HIGH‐BETA* fruit is not hydroxylated by a transgenic BCH (Giuliano *et al.*, 2008). According to this hypothesis, hydroxylation of β‐carotene in the genotypes *hp3/B^Sh^/gs* and *hp3/B^Sh^/CcBCH2* is enabled when channelling of carotenoid intermediates exist inside a presumed enzymatic complex. In this case, the tomato endogenous BCH2 may be more efficient than the transgenic CcBCH2.

Transgenic expression of BCH2 from *C. clementina* in the genetic background of *hp3/B^Sh^* raised zeaxanthin accumulation, but significantly decreased the level of β‐carotene, as well as total carotenoids content. This phenotype could be attributed to either degradation of zeaxanthin that is similar to an observation reported in Arabidopsis callus cells (Schaub *et al.*, 2018), or to co‐suppression of the endogenous tomato β‐carotene hydroxylase by the transgenic *C. clementina* BCH2. The mutation in the *white‐flower* mutant that was used in this experiment is located within the intron–exon junction of exon #2 in the tomato BCH2, which results in exon skipping during RNA processing (Galpaz *et al.*, 2006). The mechanism by which this mutation increases the zeaxanthin level in the mutant line *hp3/B^Sh^/wf/CcBCH2#1* (Table 3) is unknown. However, it can be explained by eliminating the co‐suppression of both the endogenous BCH and transgenic *C. clementina* BCH2 genes by the transgenic overexpression of CcBCH2. Although transgenic expression of citrus BCH2 in the triple mutant, line hp3/BSh/wf/CcBCH2#1, increased the amount of β‐xanthophylls nearly twofold by utilizing most of the β‐carotene, the total carotenoid concentration decreased by 59% (Table 3). Furthermore, the overall xanthophyll concentration in this mutant was higher than in the corresponding parent, *hp3*, by approximately fivefold, but was not accompanied by an accumulation of β‐carotene. This is probably the reason for the observed reduction in total fruit carotenoid content (Table 3).

A comparison between the transgenic to nontransgenic approaches in order to obtain a high‐zeaxanthin tomato shows that the latter was more successful. This consequence suggests that besides metabolic engineering by manipulation of a particular gene, accumulation of zeaxanthin is influenced by factors other than the expression of a specific carotenoid biosynthesis gene, such as plastid differentiation, as well as, chromoplast and plastid ultrastructure (Lado *et al.*, 2015). The nontransgenic high‐zeaxanthin line, which we named Xantomato, has a commercial advantage over the transgenic lines in the present market situation. However, both high‐pigment mutations that were used in this study to increase zeaxanthin accumulation, *hp3* and *hp2^dg^*, have negative pleiotropic effects on plant development and tolerance to stresses and thus have deleterious effects on yield. The rate of the effect has been difficult to measure in plants with indeterminate growth. However, the economic advantages of tomato fruit, which contains 577 μg/g DW zeaxanthin and 529 μg/g DW of β‐carotene achieved here, may overcome this adverse horticultural effect.

## Experimental procedures

### Plant material and growth conditions

Tomato (*Solanum lycopersicum*) cultivar M82 served as a reference ‘wild‐type’. The tomato mutant *HIGH‐BETA* (allele *B^Sh^*) plant was isolated by visual screening for orange fruit in a population of widespread heirloom tomato lines and was identified as a Jaune Flamme line of an unknown genetic background with a phenotype of indeterminate growth and orange‐coloured fruit. The mutants *high‐pigment 3* (*hp3‐1*) (e1472) and *white‐flower 1* (*wf1‐2*) (e1827) were previously described (Galpaz *et al.*, 2006; Galpaz *et al.*, 2008). The mutant *green‐stripe* (*gs*) was originated from line LA0212 (Tomato Genetics Resource Center, Davis; https://tgrc.ucdavis.edu/Monogenic%20stock%20list‐2014.pdf) and was found in an heirloom tomato line of unknown background. Mutants *green‐flesh* (*gf*) ‘*Nyagous*’ cultivar and *high‐pigment 2* (*hp2^dg^*) were obtained from the collection of Prof. Zamir (The Hebrew University of Jerusalem, Israel)**.**

Plants were grown in the greenhouse for crossings as described (Neuman *et al.*, 2014). Phenotypic characterization was verified over four growing seasons in plants grown in greenhouses and open fields. The transgenic 35S:CRTI in the tomato line Ailsa Craig (Romer *et al.*, 2000) were kindly provided by Prof. Paul D. Fraser (Royal Holloway, University of London).

### Pigment extraction and analysis

Fresh samples of fruit and leaves were collected from three biological replicates. Leaf pigments were extracted from 500 mg of young leaves, incubated with acetone overnight in Eppendorf tubes. The tissue was ground and, following centrifugation, the acetone phase was dried under a stream of N_2_. Fruit pigments were extracted from 150 to 250 mg of fresh pericarp tissue of fruit at the 'mature‐green' or 'ripe' stages. Pericarp tissue of Xantomato fruit at the ripe stage contains 93.1 ± 0.5% water. The tissue was ground in 1 mL of 1:1 water chloroform mixture. The chloroform phase was separated by centrifugation and dried under a stream of N_2_. The dried carotenoid extracts were dissolved in 500 μL acetone. Carotenoids were separated by high‐performance liquid chromatography (HPLC) using a Waters system consisting of a Waters 600 pump, Waters 996 photodiode array detector and Waters 717 plus auto sampler (Waters, Milford, MA). The static phase consisted of Spherisorb^®^ ODS2 C18 reversed‐phase column from Phenomenex (silica 5 μm, 3.2 mm 250 mm) (Phenomenex^®^, Torrance, CA) and the mobile phase consisted of a solvent gradient as described in Table S1 at a constant flow of 1.6 mL/min (Table S1). The spectrum between 200 and 700 nm was recorded at a rate of one full spectrum per second. The carotenoids were identified according to their typical retention time certified by standards and characteristic absorption spectra.

### DNA extraction and genotyping using DNA markers

Young tomato leaf samples of approximately 15 mg were used for DNA extraction as described previously (Eshed and Zamir, 1995). The genotyping of the homozygous mutants was confirmed by Cleaved Amplified Polymorphic Sequence (CAPS) or by sequencing. Primers and CAPS restriction enzymes for genotyping are described in Table S2.

### RNA extraction and measurement of mRNA with Quantitative Real‐Time RT‐PCR

RNA extraction from fruit at the ‘ripe’ stage was performed of approximately 200 mg powder tissue with TRI Reagent RNA isolation reagent (Sigma‐Aldrich) according to the manufacturer’s protocol. Reverse transcription and DNase treatment were performed with the iScript™ gDNA Clear cDNA Synthesis Kit #172‐5035 (Bio‐Rad). To eliminate genomic DNA contamination, the cDNA was amplified using ACTIN primers (Table S3) that differentiate between genomic DNA and cDNA sequences. The cDNA was amplified by ProFlex PCR System (Applied Biosystems by Thermo Fisher Scientific). Quantitative polymerase chain reactions were performed using the Applied Biosystems™ Fast SYBR™ Green Master Mix on a StepOnePlus™ Real‐Time PCR System (Applied Biosystems). Cycling conditions were 95 °C for 20 s, followed by 40 cycles of 95 °C for 3 s, 60 °C for 30 s and fluorescence acquisition at 60 °C. For each gene, the relative mRNA level was determined in three biological replicates. The gene ACTIN served as a control for normalization. Primers used for RT‐PCR amplification are described in Table S3.

### Expression of *BCH2 from C. clementina *in *E. coli*

The cDNA of β‐carotene hydroxylase 2 (BCH2) from *Citrus clementina* (NCBI accession number: txid85681) was obtained from RNA isolated from pulp of fresh fruit followed by RT‐PCR using primers 5′‐AGCC**ACTAGT**TGCCCGCGTGGCCGAGAAATTG‐3′ (forward) and 5′‐GTCG**CTCGAG**CTGATCCAAAAATTGGTCCTC‐3′ (reverse). For expression in *E. coli*, 267 nucleotides were truncated from the 5′ end of the predicted mRNA (NCBI Reference Sequence: XM_006421968.1), which includes part of the presumed transit peptide. The remaining cDNA sequence from nucleotide 267 to 936 was cloned into the plasmid pBluescript SK^+^ between the SpeI and XhoI sites, generated pBCH2 vector. The insert was sequenced to identify possible PCR‐derived mutations. The plasmid was transfected to *E. coli* strain XL1‐Blue grown on Luria–Bertani (LB) containing plasmid pAC‐BETAipi, which carries *Erwinia herbicola ipi, crtE, crtB, crtI, crtY* was used (Cunningham and Gantt, 2007). To enhance the expression of BCH2, 24 mg/L of isopropyl 1‐thio‐β‐D‐galactopyranoside (IPTG) were added to the LB medium. *E. coli* cells were grown overnight at 37 °C on LB solid plates followed by 5 days at room temperature for pigment accumulation.

### Construct design and tomato transformation

Full‐length cDNA of *BCH2* from *C. clementina* fruit tissue was used as template for PCR amplification using the following primers: 5′‐CCACAATCCACAATCCACTTC‐3′ (Forward) and 5′‐TGATCCAAAAATTGGTCCTC‐3′ (Reverse) and cloned into the multiple cloning site of the plasmid pJET 1.2 (CloneJET PCR Cloning Kit # K1231) (Thermo Fisher Scientific, Eldan Electronic Instruments Ltd. Israel). The insert was sequenced to confirm its integrity and was excised from the plasmid by the restriction enzymes XhoI and XbaI. The insert was cloned into the XhoI and XbaI sites of the intermediate plasmid pART7 behind the CaMV constitutive promoter 35S, with a Nos terminator, and then cleaved by the restriction enzyme NotI and ligated into the T‐DNA binary vector pART27, which contains the NPTII gene for kanamycin resistance (Gleave, 1992). This plasmid was designated pBCH2‐pART27. The plasmid pBCH2‐pART27 was transfected into *Agrobacterium tumefaciens* (GV3101) by electrophoresis. Transformed *Agrobacterium* culture was incubated for 15 min with detached 10‐day‐old tomato cotyledons of the homozygous double‐mutant *hp3/B^Sh^*, obtained in F_2_ progenies of a cross between the *high‐pigment 3* (*hp3*) (Galpaz *et al.*, 2008) and *HIGH‐BETA B^Sh^* (this work). Cotyledons were placed on feeder plates and after 48 h were transferred to an SL1 medium (Jones medium with 400 mg/L carbenicillin, 1 mg/L zeatin, 100 mg/L kanamycin and 0.7% agar). After 14 days, the cotyledons were transferred to an SL2 medium (Jones medium with 250 mg/L carbenicillin, 100 mg/L Kanamycin, 1 mg/L zeatin, 0.5 mg/L zeatin riboside, 0.1 mg/L IAA and 0.8% agar). Calli were excised from the cotyledons after a week or so and transferred again to SL2 medium plates. The calli were then transferred to an SL3 medium (Jones medium with 250 mg/L carbenicillin, 70 mg/L Kanamycin, 0.15 mg/L zeatin, 0.1 mg/L IAA and 0.8% agar). Shoots emerging from the calli were transferred to rooting medium (Nitsch medium with 150 mg/L carbenicillin, 50 mg/L Kanamycin, 50 mg/L Kanamycin and 1–2 mg/L IBA) for additional two weeks and then transferred to a transparent container with wet soil. Sturdy plantlets were finally transferred to 4‐litre pots in the greenhouse. Transgenic plants were identified by PCR amplification of the T‐DNA sequence (primers described in Table S2) and sequencing.

### Transmission electron microscopy (TEM)

The ultrastructures of chromoplasts in the pericarp of ripe fruit were examined using transmission electron microscopy (TEM) as described in Galpaz *et al. *(2008). Fresh tomato fruit in the ripe stage of development was hand‐cut. Samples were fixed with 5% glutaraldehyde in 0.1 m cacodylate buffer solution, pH 7.4, overnight at 4 °C. After four washes in cacodylate buffer, tissue was post‐fixed with cacodylate buffer, containing 2% OsO4 and 1.5% potassium ferricyanide, for 2 h at room temperature, dehydrated in a graded ethyl alcohol series followed by propylene oxide and embedded in Agar 100 resin (Agar Scientific; http://www.agarscientific.com). Polymerization was carried out at 60 °C for 48 h. For electron microscopy, ultrathin sections were cut on an LKB III ultratome. Ultrathin sections were mounted on copper 200 mesh thin bar grids and stained with uranyl acetate and lead citrate. Images were captured using a Tecnai‐12 electron microscope (Philips; http://www.philips.com) equipped with a Megaview II CCD camera and analysis software version 3.0 (Soft Imaging System GmbH).

## Conflict of interest

The authors declare no conflict of interest.

## Author contribution

UK and JH conceived the idea and designed the research plan. UK carried out the genetic manipulations to generate the *hp3/B^sh^* genotypes as well as the transgenic manipulations and analysed the carotenoids. AK and DZ performed the breeding with *hp2^dg^* and carried out the field tests. The manuscript was written by UK and edited by JH and DZ.

## Supporting information


**Figure S1** Concentration of zeaxanthin + β‐cryptoxanthin in ripe fruits from various genotypes (see text for details).
**Figure S2** Typical fruit of *green‐stripe* (*gs*) and Xantomato.
**Figure S3** Concentration of zeaxanthin plus β‐cryptoxanthin in ripe fruits of various genotypes.
**Table S1** The solvent gradient procedure used for the separation of carotenoids by HPLC at a constant flow of 1.6 ml/min.
**Table S2** The primer used for the genotyping of the different tomato mutants.
**Table S3** Primer used in quantitative RT‐PCR amplification of tomato genes.
