# Supplementary material for: Development of zeaxanthin‐rich tomato fruit through genetic manipulations of carotenoid biosynthesis
Source: Plant Biotechnol J. 2020 May 11;18(11):2292–303. doi: 10.1111/pbi.13387 (PMC7589248; doi:10.1111/pbi.13387)
Supplement: Supplementary file 1 — Figure S1 Concentration of zeaxanthin + β‐cryptoxanthin in ripe fruits from various genotypes (see text for details). Figure S2 Typical fruit of green‐stripe (gs) and Xantomato. Figure S3 Concentration of zeaxanthin plus β‐cryptoxanthin in ripe fruits of various genotypes. Table S1 The solvent gradient procedure used for the separation of carotenoids by HPLC at a constant flow of 1.6 ml/min. Table S2 The primer used for the genotyping of the different tomato mutants. Table S3 Primer used in quantitative RT‐PCR amplification of tomato genes. [file PBI-18-2292-s001.docx]

**Supporting Information**

**Table S1**: The solvent gradient procedure used for the separation of carotenoids by HPLC at a constant flow of 1.6 ml/min.

| Time (minutes) | Acetonitrile:H_2_O (9:1) | Ethyl acetate |
| --- | --- | --- |
| 0-8 | 100% | 0% |
| 8-12 | 80% | 20% |
| 12-26 | 65% | 35% |
| 26-26.1 | 45% | 55% |
| 26.1-33 | 0 | 100% |

**Table S2**. The primer used for the genotyping of the different tomato mutants

| **Mutant name** | **symbol** | **Gene** | **Forward** | **Reverse** | **Type** | **Restriction enzyme** |
| --- | --- | --- | --- | --- | --- | --- |
| *HIGH-BETA* | *B^Sh^* | *CycB* | 5’- AGGGTTGTCAAAAATGTCTCA-3’ | 5’- AAAAGGTAATTTACTGAGTTGTGCAT-3’ | length polymorphism (43 bp insertion) |  |
| *high-pigment 3* | *hp3* | *ZEP* | 5’-AACCCACAAATCCCACTTTC-3’ | 5’-TTCTCTTCGGACAAGCACAC-3’ | CAPS | DraI |
| *GREEN FLESH* | *gf5* | *SGR1* | 5’-CTCGATTTCAATTTCCTTCAGC-3’ | 5’-CCATCCTAAACTTGATGTTCTTGTC-3’ | length polymorphism (1164 bp deletion) |  |
| *Green-stripe* | *gs* | unknown |  |  | phenotypic |  |
| *white-flower* | *wf* | *CrtR-b2* | 5’-ATCTTGTGGCAGCTGTGATG-3’ | 5’-TGACCTCCAACTTTCATAATGC-3’ | sequencing |  |
| *C. clementina β-carotene hydroxylase* | *CcBCH2* | *BCH2* | 5’-CTATCCTTCGCAAGACCCTTCC-3’  (35S) | 5’-TGATCCAAAAATTGGTCCTC-3’ | dominant (transgenic) |  |
| *CrtI* | *CrtI* | *CrtI* | 5’-CTATCCTTCGCAAGACCCTTCC-3’  (35S) | 5’-GCAAAACTTTTCGAGCCAAC-3’ | dominant (transgenic) |  |
| *high-pigment 2* | *hp2dg* | *DET1* | 5’-TTCTTCGGATTGTCCATGGT-3’ | 5’- CACCAATGCTATGTGCCAAA-3’ | CAPS | AclI |

**Table S3:** Primer used in quantitative RT-PCR amplification of tomato genes

| **gene** | **Forward** | **Reverse** |
| --- | --- | --- |
| *CycB* | 5’-GTTATTGAGGAAGAGAAATGTGTGAT -3’ | 5’- TCCCACCAATAGCCATAACATTTT-3’ |
| *PSY1* | 5’- AACTTGTTGATGGCCCAAAC-3’ | 5’- CTGTATCGGACAAAGCACCA-3’ |
| *PDS* | 5’- TGCAATGGAAGGAACATTCA-3’ | 5’- ATTGCTGGCAAGAGTCCAAT-3’ |
| *ZDS* | 5’- CATGTCAAAGGCCACTCAGA-3’ | 5’- ACGGTAACAACAGGCACTCC-3’ |
| *ACTIN* | 5′-TTGCTGACCGTATGAGCAAG-3′ | 5′-GGACAATGGATGGACCAGAC-3′ |

**Figure S1**: Concentration of zeaxanthin + β-cryptoxanthin in ripe fruits from various genotypes (see text for details).

**
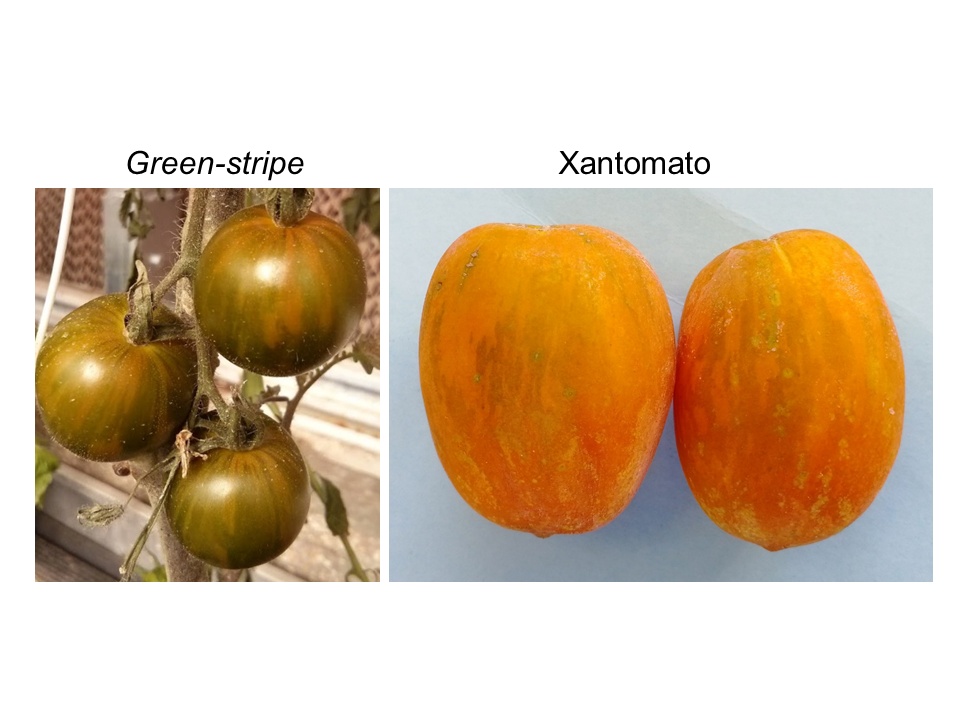
**

**Figure S2**: Typical fruit of *green-stripe* (*gs*) and Xantomato.

**Figure S3**: Concentration of zeaxanthin plus β-cryptoxanthin in ripe fruits of various genotypes. *hp3*, *high-pigment 3*, *wf*, *white-flower*; Bsh, *HIGH-BETA* allele *B^sh^*; CcBCH#1, transgenic lines expressing the *C. clementina* BCH2 (μg.g^-1^ FW).
